# Associations of psychosocial factors, knowledge, attitudes and practices with hospitalizations in internal medicine divisions in different population groups in Israel

**DOI:** 10.1186/s12939-021-01444-z

**Published:** 2021-04-20

**Authors:** Shira Sagie, Wasef Na’amnih, Juda Frej, Gershon Alpert, Khitam Muhsen

**Affiliations:** 1 Department of Epidemiology and Preventive Medicine, School of Public Health, Sackler Faculty of Medicine, Tel Aviv University, Ramat Aviv, 6139001 Tel Aviv, Israel; 2 Department of Oncology, Sheba Medical Center, 52621 Ramat Gan, Israel; 3 Clalit Health Services, Hadera, Israel

**Keywords:** Health-disparities, Hospitalizations, Psychosocial factors, Social determinants

## Abstract

**Background:**

Inequalities in healthcare utilization exist across ethnic groups; however, the contributions of health-related knowledge and psychosocial factors to these inequalities remain unclear. We examined associations of social determinants of health, psychological factors, knowledge, attitudes and health practices, with hospitalizations in internal medicine divisions, among Israeli adults, Jews and Arabs, with non-communicable diseases, in a setting of universal health insurance.

**Methods:**

A retrospective study was undertaken among 520 Jews and Arabs aged 40 years or older with non-communicable diseases, members of a large health maintenance organization. Hospitalization (at least once during 2008) in an internal medicine division was determined based on documentation in electronic health records. Participants were randomly selected in strata of sex, population-group and hospitalization status (yes/no). Data were collected from medical records and via face-to-face interviews using a structured questionnaire. Main independent variables included comorbidity burden, health behaviors, mental health wellbeing and self-rated health. Scales measuring health knowledge and attitudes/beliefs were constructed using factor analysis.

**Results:**

Comorbidity burden (OR 1.41 [95% CI 1.24–1.61]) and self-rated health (not good vs. good) (OR 1.88 [95% CI 1.13–3.12]) were positively associated with hospitalizations in an internal medicine division, while an inverse association was found with better mental health wellbeing (OR 0.98 [95% CI 0.96–0.99, for each 1-point score increase). Among Jewish participants, positive associations were found of the number of offspring, comorbidity burden and perceived difficulty, with hospitalizations. No significant associations were found with hospitalizations of other sociodemographics, health behaviors, knowledge and attitudes/beliefs.

**Conclusions:**

Comorbidity burden was the main risk factor of hospitalizations in internal medicine divisions. Psychosocial factors, such as self-rated health, a complex variable affected by social capital, mental wellbeing, the number of offspring, and perceived burden and difficulty, seem also to contribute. These findings suggest the involvement of broad family and social factors, beyond individual level characteristics and medical needs, in hospitalizations in internal medicine divisions. Interventions to reduce hospitalizations should be comprehensive and integrate aspects of mental health wellbeing; they should build on familial characteristics (e.g., number of offspring), factors related to social capital such as self-rated health, and perceived burden and difficulty.

**Supplementary Information:**

The online version contains supplementary material available at 10.1186/s12939-021-01444-z.

## Background

Disparities have been described between ethnic groups in health indicators including: non-communicable disease burden and risk factors [1–12]. These disparities may be partially explained by social determinants [7, 9–13]. Health behaviors such as smoking and physical inactivity might also contribute to inequalities in non-communicable diseases [8–11, 14]. Moreover, socioeconomic status (SES) and ethnic differences in access to care and healthcare utilization have been reported [9, 15–24], and these might contribute to disparities in relation to non-communicable diseases [9].

In the United States, ethnic differences in access to and in utilization of healthcare services have been diminished following the implementation of the Affordable Care Act reform [18, 19]. For migrant populations in Europe, lower utilization of healthcare services has been reported compared to non-migrants [21, 22, 24, 25], specifically lower utilization of screening tests and outpatient visits for specialized care. However, hospitalizations and emergency room visits were more common among migrants than non-migrants [25]. Such differences were partially explained by financial difficulties [21, 22, 24]. The cost of hospitalization is greater than outpatient visits [26] and comprises the main driver of treatment costs. Therefore, understanding factors that might affect utilization of hospitalizations is warranted. Age was positively related to hospitalizations, while being unmarried and receiving support from friends were inversely related to hospitalizations [27]. Interestingly, comorbidity burden was not a major determinant of hospitalization in that study. The risk factors for hospitalization among persons aged 85 years or more in Germany were functional decline, depression, higher social networks and chronic conditions [28]. An analysis of high-risk veteran affairs patients in the United States, which considered patient-reported social and behavioral determinants of health, better predicted the risk of hospitalization than did information only available in electronic health care records [29]. The social determinants examined in that study included marital status, resilience, smoking and health literacy. Despite this evidence, the contribution of individual level social determinants, psychosocial factors and health behaviors to the risk of hospitalizations in persons with non-communicable diseases remains elusive. Understanding the role of these factors can help identify new risk groups and modifiable factors for hospitalizations. In Israel, a country with universal health insurance, differences in utilization of healthcare services have been reported between Jews, who comprise about 75% of the population, and Arabs, the main ethnic minority, comprising about 21% of the population [20, 30, 31]. Visits to primary care physicians and the emergency room, and hospitalizations were shown to be more common among Arabs than Jews [20, 30, 31]. However, visits to specialist physicians were less common among Arabs than Jews [20]. Given the socioeconomic and cultural diversity between the Arab and Jewish populations, the aim of this study was to examine associations of social determinants of health, psychological factors, knowledge, attitudes and health practices, with hospitalizations in internal medicine divisions, among Arabs and Jews with non-communicable diseases.

## Methods

### Study population and design

This retrospective study was conducted based on a cross-sectional study of 28,393 individuals [30], Jews and Arabs, aged 40 years or above, with cardiovascular disease (CVD), hypertension or diabetes, residents of the Hadera sub-district, utilizing the database of the Clalit Health Services, the largest health maintenance organization in Israel.

The population in Hadera sub-district includes Jewish residents and Arab residents, mostly Muslims. During 2008, the population in Hadera sub-district included 347,600 residents, of whom 183,400 (52.8%) were Jews, 155,100 (44.6%) Arabs and 2.6% belonged to other ethnicities [32]. The Jewish population includes individuals who were born in Israel and Jews who immigrated to Israel from other countries. Arab residents generally live in separate towns and villages from the Jewish residents. Israeli residents have universal healthcare insurance according to the National Health Insurance Law implemented since 1995 [33]. Access to care is universal and covers outpatient and inpatient services. Primary care clinics are available in each town, and Arabic speaking medical staff usually work in Arab towns. Hospitals and large specialists’ clinics are typically located in Jewish cities.

### Sampling method and sampling frame

The sample of the current study was randomly selected from individuals who were included in the above mentioned study [30]. During 2008, the database of Clalit Health Services included records of 31,883 individuals aged 40 years or older with documented CVD, hypertension or diabetes. After excluding individuals with cancer diagnoses (*n* = 3471) and those without information on ethnicity (*n* = 19), data of 28,393 individuals were analyzed [30]. Of these, 3516 (12.4%) were hospitalized at least once in an internal medicine division in 2008, based on documentation in the Clalit database [30]. An analysis has been published of correlates of hospitalizations of the entire cohort, based on data available in electronic health records [30]. This included associations of visits to the emergency room, visits with specialist physicians and uptake of screening tests, with hospitalizations. More details on emergency room and specialist physicians by population and sex group are presented in Additional file 1.

A data analyst at Clalit Health Services randomly selected 750 individuals from the above mentioned dataset [30], in strata of sex, population group (Jewish or Arab) and status of hospitalization in an internal medicine division in 2008 (yes or no) (Additional file 2).

### Data sources and data collection tool

Registered nurses working in the primary care clinics of the selected individuals called them and offered participation in the study. Overall, 520 (70%) of 750 individuals agreed to participate and signed written informed consent. Compliance was 60% among Jews and 80% among Arabs. Data were collected via face-to-face interviews conducted during 2009 (about 1 year after the hospitalizations) in the participants’ language (Hebrew or Arabic) by study nurses who received standardized training. Three persons who did not speak Hebrew or Arabic were excluded from the study.

Data on comorbidities and hospitalizations were collected from electronic health records, as described elsewhere [30]. Data on socioeconomic factors, subjective health status, psychological health, knowledge, attitudes and health practices, and potential barriers towards usage of healthcare services were collected via personal interviews using a structured questionnaire (Additional file 3). The questionnaire included items that were specifically constructed for this study and items that were used in national and international surveys [34–36]. The questionnaire comprised multiple question formats, including closed-ended and open-ended questions, and questions that used the Likert scale. Validation of the questionnaire included assessment of face and content validity. Convergent and construct validity of various items was assessed, in addition to alpha Cronbach measurement of internal consistency of the constructed scales.

### Definition of the study variables

The dependent variable was defined as at least one hospitalization in internal medicine divisions during the study period (yes or no), based on the documentation in the database of Clalit Health Services. An internal medicine division included: internal medicine departments, intensive care units, cardiac intensive care units and neurology [30].

### The independent variables

Detailed operational definitions of the independent variables are described in Additional file 4. These variables were selected based on evidence that they might have a role in health outcomes and utilization of healthcare services [13, 18, 27–30, 37–39].

#### Sociodemographic variables

These included sex, age (a continuous variable, in years), population group (Arabs/Jews), marital status (dichotomous variable: married or cohabiting; and unmarried [single, widowed or separated/divorced]), number of offspring (discrete variable), household monthly income (dichotomous variable: above or below 3600 New Israeli Shekels), and number of schooling years (a continuous variable). Religiosity was defined according to the participants’ answer to a single question: “How do you define yourself?” The possible responses were orthodox (very religious), religious, traditional and secular. The responses were grouped into two categories: religious (orthodox/religious) vs. traditional/secular. Household density index was calculated as the number of people living in the household divided by the number of rooms.

For Jewish participants, country of birth was determined by participants’ self-report, and classified as Israel, Asia/Africa or Europe/America/Russia.

#### Variables of health status

Self-rated health status was classified based on the participants’ replies to the question: “Overall how is your health?” The answers were: very good, good, intermediate, bad and very bad. The variable self-rated health was analyzed as dichotomous variables (good [including very good/ good)] vs. not good [including intermediate/bad/very bad]). Self-rated health is a known predictor of mortality [37] and a widely used indicator of health. A comorbidity score was calculated using data on diagnoses retrieved from electronic health records, as described elsewhere [30].

Mental health was classified based on participants’ reports on the 5-mental health items (MHI-5 score) of the 36-item Short Form (SF36) health survey questionnaire [34–36]. The MHI-5 is a measure of current mental well-being and includes questions on depressive symptoms and nervousness. Scoring of the MHI-5 was described elsewhere [34–36]. Higher scores represent better mental health. MHI-5 has good construct validity and was shown to be useful for identifying mood and anxiety disorders [40, 41]. Alpha Cronbach of the MHI-5 in our sample was 0.895.

#### Health behaviors

Physical inactivity (a dichotomous variable, yes or no) was classified based on the participants’ reply to the question “Do you regularly engage in physical activity lasting 20 minutes or more, for example: walking, running, swimming, gymnastic exercise or ball games?”. The answers were “almost every day” “once to twice a week”, “once to twice a month”, “less than once a month” and “never”. Participants who replied “never” were classified as physically inactive.

Body mass index (BMI) was calculated based on the participants’ reports of their weight in kilograms (kg) (in light clothes and without shoes) and height in centimeters using the formula: weight (kg)/ height (m)^2^. Smoking was defined based on participants’ report of being a current smoker (yes or no [including never/past smoker]).

#### Access and barriers to healthcare services

Having a complementary health insurance, i.e. in addition to the universal basic health insurance package, was defined based on the participants’ reports (dichotomous variable, yes or no). Language barrier/ fluency in Hebrew was defined using a score that was calculated via a confirmatory factor analysis (Additional file 4) (alpha Cronbach 0.83).

#### Health related knowledge and attitudes/beliefs

Scores were created using multiple items in the questionnaire by means of exploratory factor analysis. The aim was to assess knowledge on health and lifestyle risk factors (alpha Cronbach 0.66); attitudes regarding trust in the medical system, preventive medicine (alpha Cronbach 0.67) and alternative medicine (alpha Cronbach 0.56); and beliefs in fate and superstitions (alpha Cronbach 0.61) (Additional file 4). Perceived treatment difficulties and burden were classified using a score that was calculated via an exploratory factor analysis (Additional file 4) that included two items.

### Statistical analyses

Factor analysis was used to build scores; measuring health-related knowledge and attitudes/beliefs (Additional File 4). Alpha Cronbach was used to assess internal consistency across the items that were included in these scales.

The study sample was described using medians and interquartile range (IQR) for continuous and discrete variables, and frequencies and percentages for categorical variables. Deviation from normal distribution of continuous variables was examined by histograms, Shapiro Wilk test and Kolmogorov–Smirnov test. The associations of sociodemographic, comorbidity and other independent variables with hospitalization in internal divisions were examined using the Mann-Whitney *U* test for variables that did not follow a normal distribution and the chi square test and Fisher Exact test were used as appropriate for categorical variables. A total sample analysis was performed, as well as a stratified analysis by population group. Interactions between the variable “population group” and each of the independent variables were examined using logistic regression analysis. We fitted multivariable logistic models. Adjusted OR and 95% confidence intervals (CI) were obtained for the independent variables were obtained from these models. *P* < 0.05 was considered statistically significant.

#### Handling missing data

Few variables had low proportion of missing values. The proportion of missing values was greater for income (22.6%) and having complementary health insurance (8.3%) (Additional file 5), thus, multiple imputation [42] was performed for these variables.

The statistical analyses were performed using SPSS version 25 and R version 3.4.2.

## Results

The study included 520 participants, the median age was 65 years (IQR 17). Overall, 185 (35.6%) participants were hospitalized in an internal medicine division. Arab participants were younger and had a lower educational level than Jewish participants. The proportions of participants with incomes above 3600 NIS and unmarried were lower among Arab than Jewish participants. Arab participants had more offspring, more often reported being religious and lived in more crowded households than Jewish participants. Diabetes was more prevalent and mental health scores (MHI-5) were worse among Arabs than Jews. A smaller proportion of Arabs than Jews had complementary insurance. The median BMI and smoking prevalence were higher among Arabs (Table 1). Smoking prevalence was 50.4% in Arab men and 15.4% in Jewish men, *P* < 0.001; and 6.5% in Arab women and 13.6% in Jewish women, *P* = 0.04.

**Table 1:** Characteristics of the study sample by population group

	Overall (*n* = 520)	Jews (*n* = 248)	Arabs(*n* = 272)	P
**Sociodemographic variables**				
Hospitalized, n (%)	185 (35.6)	85 (34.3)	100 (36.8)	–
Sex, women, n (%)	280 (53.8)	125 (50.4)	155 (57.0)	0.15
Age in years, median (IQR)	65.0 (17.0)	70.0 (16.0)	62.0 (17.0)	< 0.001
Country of birth, n (%)				
Israel	–	47 (19.1)	–	
Asia/Africa	–	115 (46.7)	–	
Europe/ America/Russia	–	84 (34.1)	–	
Marital status, not married, n (%)	158 (30.4)	91 (36.7)	67 (24.6)	0.004
Number of offspring, median (IQR)	4 (3)	3 (2)	6 (3)	< 0.001
Household density, median (IQR)	0.80 (0.50)	0.60 (0.29)	1.00 (0.67)	< 0.001
Monthly income above 3600 Shekels, n (%)	224 (52.8)	124 (67.0)	100 (41.8)	< 0.001
Religious, yes, n (%)	234 (45.9)	52 (21.4)	182 (68.2)	< 0.001
Number of years of schooling, median (IQR)	8.5 (8.0)	11.0 (6.0)	8.0 (9.0)	< 0.001
**Health status and health behaviors**				
Comorbidity score, median (IQR)	4.0 (2.0)	4.0 (2.0)	4.0 (2.0)	0.8
Hypertension, n (%)	389 (74.8)	190 (76.6)	199 (73.2)	0.4
Cardiovascular disease, n (%)	240 (46.2)	113 (45.6)	127 (46.7)	0.8
Diabetes, n (%)	282 (54.2)	119 (48.0)	163 (59.9)	0.009
MHI-5 score, median (IQR)	56.0 (16.0)	60.0 (12.0)	52.0 (16.0)	< 0.001
Self-rated health, not good, n (%)	356 (68.6)	166 (67.2)	190 (69.9)	0.5
Physical inactivity, n (%)	281 (54.9)	124 (50.8)	157 (58.6)	0.094
BMI, median (IQR), kg/m2	29.4 (6.9)	28.8 (6.2)	30.2 (7.3)	< 0.001
Smoking, n (%)	105 (20.2)	36 (14.5)	69 (25.5)	0.003
Complementary health insurance, Yes, n (%)	331 (69.0)	208 (85.6)	123 (51.9)	< 0.001

*P* value was obtained by the chi square test and Fisher Exact test, as appropriate, for categorical variables and Mann-Whitney *U* test for continuous and discrete variables

*BMI* body mass index, *IQR* interquartile range, *MHI-5* mental health 5-items

### Bivariate analysis

Compared to the non-hospitalized group, among individuals who were hospitalized in an internal medicine division, the mean age was older, the number of offspring greater, and the income and years of schooling lower. Among those who were hospitalized, the comorbidity burden and physical inactivity prevalence were higher, and the mental health score was lower. Self-rated health as “not good” was more common in the hospitalized group. Perceived difficulty and burden related to treatment were positively associated with hospitalization. No significant associations were found of health knowledge, attitudes and beliefs with hospitalization (Table 2). No significant interactions were found between the variable “population group” and the other independent variables, except for a borderline statistically significant interaction between “population group” and “believes in fate and superstition” (Additional file 5).

**Table 2:** Sociodemographic and health-related correlates of hospitalizations in internal medicine divisions- overall and stratified analysis

	Overall			Jews			Arabs		
Hospitalized	No	Yes	***P***	No	Yes	***P***	No	Yes	***P***
Number	335	185		163	85		172	100	
**Socio-demographic variables**									
Sex, women, n (%)	186 (55.5)	94 (50.8)	0.3	81 (49.7)	44 (51.8)	0.8	105 (61.0)	50 (50.0)	0.10
Age, median (IQR)	64.0 (17.0)	67.0 (18.0)	0.034	69.0 (14.0)	72.0 (18.0)	0.16	59.5 (18.0)	64.0 (17.0)	0.04
Country of birth, n (%)			0.2			0.3	NA	NA	
Israel	203 (61.0)	116 (62.7)		31 (19.3)	16 (18.8)				
Asia/Africa	70 (21.0)	45 (24.3)		70 (43.5)	45 (52.9)				
Europe/ America/Former Soviet Union	60 (18.0)	24 (13.0)		60 (37.3)	24 (28.2)				
Marital status, not married, n (%)	96 (28.7)	62 (33.5)	0.2	55 (33.7)	36 (42.4)	0.2	41 (23.8)	26 (26.0)	0.8
Number of offspring, median (IQR)	4.0 (3.0)	5.0 (4.0)	< 0.001	3.0 (2.0)	4.0 (4.0)	0.027	5.0 (3.0)	6.0 (4.0)	0.009
Household density, median (IQR)	0.75 (0.54)	0.67 (0.50)	0.8	0.60 (0.40)	0.67 (0.25)	0.4	1.00 (0.67)	1.00 (0.67)	0.4
Monthly income above 3600 Shekels, n (%)	198 (59.0)	85 (45.9)	0.009	118 (72.4)	48 (56.5)	0.016	79 (45.9)	37 (37.0)	0.17
Religiosity, yes, n (%)	148 (45.0)	86 (47.5)	0.6	32 (20.1)	20 (23.8)	0.6	116 (68.2)	66 (68.0)	1.0
Years of schooling, median (IQR)	9.0 (7.0)	8.0 (12.0)	0.003	11.0 (6.0)	10.0 (10.0)	0.008	8.0 (9.0)	7.0 (9.0)	0.16
**Health status and health behaviors**									
Comorbidity score, median (IQR)	3.0 (1.0)	5.0 (3.0)	< 0.001	3.0 (1.0)	4.0 (1.5)	< 0.001	3.0 (2.0)	5.0 (3.0)	< 0.001
MHI-5 score, median (IQR)	60.0 (14.5)	52.0 (16.0)	< 0.001	60.0 (16.0)	56.0 (20.0)	< 0.001	56.0 (16.0)	50 (20.0)	0.001
Self-rated health status, not good, n (%)	201 (60.0)	155 (84.2)	< 0.001	98 (60.1)	68 (81.0)	0.002	103 (59.9)	87 (87.0)	< 0.001
Physical inactivity, n (%)	156 (47.4)	125 (68.3)	< 0.001	68 (42.5)	56 (66.7)	0.001	88 (52.1)	69 (69.7)	0.007
BMI, median (IQR)	29.1 (6.6)	29.4 (7.6)	0.2	28.4 (6.0)	28.9 (6.5)	0.4	30.1 (7.1)	30.4 (7.7)	0.6
Smoking, n (%)	60 (17.9)	45 (24.5)	0.097	23 (14.1)	13 (15.3)	0.9	37 (21.5)	32 (32.3)	0.068
**Access and barriers to healthcare services**									
Complementary health insurance, Yes, n (%)	231 (68.9)	122 (65.9)	0.4	143 (88.7)	68 (80.0)	0.085	88 (51.1)	54 (54.0)	0.6
Low Hebrew fluency, needs translation in medical interaction, n (%)	152 (45.4)	84 (45.4)	0.9	95 (58.3)	52 (61.3)	0.6	57 (33.1)	32 (32.0)	0.8
Perceived difficulty & burden, n (%)	148 (44.2)	112 (60.5)	< 0.001	68 (41.7)	51 (60.0)	0.009	80 (46.5)	61 (61.0)	0.029
**Health knowledge and attitudes/ beliefs**									
Knowledge in health and lifestyle risk factors, median (IQR)	0.3 (0.5)	0.3 (0.8)	0.4	0.3 (0.5)	0.2 (1.0)	0.3	0.3 (0.4)	0.4 (0.5)	0.7
Trust in medical system and importance of prevention (%)	168 (50.1)	92 (49.7)	1.0	92 (56.4)	47 (55.3)	0.9	76 (44.2)	45 (45.0)	0.9
Believes in alternative medicine, n (%)	175 (52.2)	85 (45.9)	0.2	97 (59.5)	50 (58.8)	1.0	78 (45.3)	35 (35.0)	0.12
Believes in fate & superstition, n (%)	167 (49.9)	93 (50.3)	1.0	59 (36.2)	40 (47.1)	0.12	108 (62.8)	53 (53.0)	0.14

*BMI* body mass index, *IQR* interquartile range, *NA* Not applicable, *MHI-5* mental health 5-items. *P* values were obtained by the chi square test and Fisher Exact test where appropriate for categorical variables and Mann-Whitney *U* test for continuous and discrete variables

### Multivariable analysis

An overall multivariable analysis that included both Arab and Jewish participants showed positive associations of comorbidity burden and self-rated health as “not good”, with hospitalizations in an internal medicine division (Table 3). An inverse association was found between better mental health and hospitalization. No significant associations were found of the variables: age (*P* = 0.8), sex (*P* = 0.10), income (*P* = 0.6) and the number of schooling years (*P* = 0.5), with hospitalizations (Additional file 5). In an additional model, we introduced an interaction term between the variables “population group” and “believes in fate and superstition”, which was not significant (*P* = 0.6) (Additional file 5).

**Table 3:** Overall multivariable logistic regression analysis of the correlates of hospitalizations in internal medicine divisions

	Adjusted OR [95% CI]	***P***
Population group (Jews vs. Arab)	0.83 [0.53–1.28]	0.4
Number of offspring	1.07 [0.99–1.16]	0.08
Comorbidity score	1.41 [1.24–1.61]	< 0.001
Physical inactivity	1.36 [0.89–2.09]	0.2
Perceived difficulty and burden	1.21 [0.97–1.50]	0.09
Self-rated health (not good vs. good)	1.88 [1.13–3.12]	0.02
MHI-5 score	0.98 [0.96–0.99]	0.005

*CVD* cardiovascular disease, *MHI-5* mental health 5-items. Model *R*^2^ Nagelkerke = 0.23

Among Jewish participants, positive associations were found of the number of offspring, comorbidity burden and perceived difficulty, with hospitalization in internal medicine divisions (Table 4). An inverse association was found between mental health score and hospitalization. Among Arab participants, comorbidity score and self-rated health as “not good” were positively related to hospitalization (Table 5).

**Table 4:** Multivariable logistic regression analysis of the correlates of hospitalizations in internal medicine divisions – Jewish participants

	Adjusted OR [95% CI]	***P***
Number of offspring	1.15 [1.01–1.31]	0.04
Physical inactivity	1.54 [0.83–2.88]	0.2
Perceived difficulty and burden	1.40 [1.02–1.94]	0.04
Comorbidity score	1.41 [1.15–1.71]	0.001
MHI-5 score	0.96 [0.94–0.99]	0.006

*CVD* cardiovascular disease, *MHI-5* mental health 5-items. Model *R*^2^ Nagelkerke = 0.25

**Table 5:** Multivariable logistic regression analysis of the correlates of hospitalizations in internal medicine divisions – Arab participants

	Adjusted OR [95% CI]	***P***
Perceived difficulty and burden	1.04 [0.77–1.41]	0.3
Physical inactivity	1.34 [0.74–2.46]	0.3
Self-rated health (not good vs. good)	2.81 [1.35–5.83]	0.006
Smoking	1.72 [0.91–3.21]	0.09
MHI-5 score	0.98 [0.96–1.01]	0.1
Comorbidity score	1.42 [1.19–1.69]	< 0.001

*CVD* cardiovascular disease, *MHI-5* mental health 5-items. Model *R*^2^ Nagelkerke = 0.23

## Discussion

In this study we examined associations of social determinants of health, psychological factors, knowledge, attitudes and health practices, with hospitalizations in internal medicine divisions. The cohort comprised adults with non-communicable diseases from ethnically and culturally diverse populations in Israel.

Comorbidity burden was the main risk factor of hospitalizations in internal medicine divisions among both the Arab and Jewish participants. Self-rated health as “not good” was positively associated with hospitalizations in internal medicine division, while better mental health was inversely associated with hospitalizations. Although we found no significant interactions between the variables “population group” and any of the independent variables, the significant correlates of hospitalizations in internal medicine divisions slightly differed between the Arab and Jewish participants. The number of offspring and perceived burden and difficulty were associated with hospitalizations in an internal medicine division, among Jews; while among Arab participants, self-rated health as “not good” was positively associated with hospitalizations.

Our study included socially diverse groups. Arab and Jewish participants differed significantly in socioeconomic indicators. Nonetheless, in contrast with other reports [15, 19], associations of social determinants such as income and number of schooling years with hospitalizations were not significantly different in multivariable models. This discrepancy between our data and others might be explained by different healthcare systems, namely in Israel, all citizens have health insurance and access to care is relatively high. Thus, in such setting, individuals’ need for hospitalization services are met regardless of their income or educational level. Moreover, as demonstrated in our study, the likelihood of hospitalization in an internal medicine division was largely affected by the individual’s health status (e.g., comorbidity score, mental health wellbeing). While we found no significant associations between social determinants and hospitalizations in the internal medicine division, the impact of these determinants on health care utilization is well recognized [9, 15–24].

We found that each one-point increase in MHI-5 score, i.e. better mental health wellbeing, was associated with a 2% reduced risk of hospitalization. This finding is in agreement with reports linking mental health wellbeing with various non-communicable diseases [43–45]. For example, a prospective study from China showed that a poorer mental health status (lower scores in MHI-5) was strongly associated with arrhythmia recurrence after catheter ablation of atrial fibrillation [43]. A prospective study from Denmark showed increased risk for CVD events and all-cause mortality in relation to poorer mental health wellbeing (low MHI-5 score) at baseline [44]. In a national health survey, we also found that poorer mental health status was related to being physical inactive [34]. Together these findings highlight the need for increasing attention for mental health needs and status in the primary care setting, and for strengthening mental health services, to possibly reduce excess risk for health impairment and healthcare costs.

Self-rated health is a well-established predictor of mortality [37]. A recent study from 11 European countries showed an association of self-rated health with utilization of long-term care services in a frail population [46]. Our study adds a new dimension, by showing a positive association of perceived health status as “not good” with hospitalizations. This suggests the impact of personal subjective health measures, rather than only objective medical needs, on healthcare utilization and costs. Better self-rated health was shown to be related to social capital [47, 48]; this implies the indirect involvement of social factors in healthcare seeking behaviors and costs.

Interestingly, among Jewish participants, the number of offspring was associated with an increased likelihood of hospitalizations. The number of offspring in the Jewish sample was negatively correlated with the number of schooling years (Spearman’s correlation coefficient − 0.35, *P* < 0.001) and positively correlated with religiosity (Spearman’s correlation coefficient 0.38, *p* < 0.001). This confirms the current demographic structure of the Jewish population in Israel [49]. Replacing the number of offspring by the variables education or religiosity in multivariable models, or adjusting for these two variables, did not change the results. Moreover, religiosity (*P* = 0.2) and the number of schooling years (*P* = 0.6) were not significantly associated with hospitalizations. Religiosity was shown to be related to a lower risk of mortality [50, 51] and telomere length, a known marker of longevity [52]. Nonetheless, the number of offspring was shown to be associated with an elevated risk for hospitalizations, but lower risk of mortality in an elderly cohort from Sweden [53]. Emerging evidence suggests associations of higher educational level and SES of offspring, with reduced risk of mortality of their parents [53, 54]. Collectively, our and others’ findings suggest the involvement of broad family and social factors, beyond individual level characteristics, in hospitalizations. In this study, we did not have information on the educational level and SES of the offspring. Therefore, future studies are warranted to explore the role of social determinants of offspring in healthcare utilization and health outcomes.

Significant positive associations were found of perceived difficulty and burden with hospitalization in the internal medicine division, only among Jewish participants. Such perception might reflect real functional difficulties and complicated medical conditions that increase the odds of hospitalizations. Future studies are needed to further explore underlying conditions and mechanisms that might explain such associations. Other than perceived difficulty and burden, none of the other variables related to health attitudes and beliefs was significantly related to hospitalizations.

Our study has several strengths. We comprehensively assessed potential correlates of hospitalizations in the internal medicine division, a costly health service, among adults with non-communicable diseases. The setting of a well-defined geographic region and universal health insurance eliminated numerous barriers to care. The inclusion of diverse ethnicities and SES segments increase the generalizability of our findings. We assessed medical and social determinants, as well as psychosocial factors, health knowledge, and attitudes and beliefs. Information sources included both electronic medical records and individuals’ reports on these variables. The interviews were conducted face-to-face by trained nurses in the participants’ native language. We received important information regarding the patients’ beliefs, health knowledge and detailed socioeconomic variables, which could not be obtained otherwise. We used validated scales when available, and constructed new ones in the framework of this study; fair alpha Cronbach values were demonstrated (Additional file 4). These scales can be employed and improved in future studies in other populations.

Our study also has limitations. Information on income was missing for nearly 22%, this issue was handled with multiple imputation. Other than that, missing information was overall low. Reporting bias might exist with regard to information that was obtained via interviews, however such bias is likely non-differential. Though our data were collected about one decade ago, our findings are still relevant. The study variables – health service utilization, beliefs and behaviors – generally remain stable. Moreover, we compared the variables between hospitalized and non-hospitalized groups. Therefore, if any change occurred over time in the independent variables, it would presumably occur equally in both groups. Accordingly, the period of data collection is not expected to affect the study findings. Health inequalities, including in relation to healthcare utilization and hospitalizations [31, 55] still exist between Arabs and Jews in Israel [6, 56]; and across ethnic and SES groups in numerous countries [9, 15–24]. Therefore, our findings remain important and add knowledge of the involvement of psychosocial characteristics in the hospitalization of persons with non-communicable diseases in internal medicine divisions.

## Conclusions

Our study provides preliminary evidence of associations of multiple psychosocial factors of persons with non-communicable disease, with hospitalizations in internal medicine divisions. These factors may go beyond medical needs. Further studies are needed to elucidate the mechanisms by which such factors interact with medical and behavioral factors. Comorbidity burden was the main risk factor of hospitalizations in internal medicine divisions. Psychosocial factors, such as self-rated health, a complex of variables related to social capital and mental wellbeing, also seem to contribute. Among Jews only, the number of offspring, and perceived burden and difficulty, were positively associated with hospitalizations in the internal medicine division. Collectively, our findings suggest the involvement of broad family and social factors, beyond individual-level characteristics and medical needs, in hospitalizations in internal medicine division. Interventions to reduce hospitalizations should be comprehensive and integrate aspects of mental health wellbeing; they should build on familial characteristics (e.g., the number of offspring), factors related to social capital such as self-rated health, and perceived burden and difficulty.

## Supplementary Information

**Additional file 1.**

**Additional file 2.**

**Additional file 3.**

**Additional file 4.**

**Additional file 5.**

## Data Availability

Due to local laws and regulations, individual level dataset from this study cannot be made publicly available. Additional results/ analyses/ information can be provided by the corresponding author on reasonable request.

## Abbreviations

BMI: Body Mass Index
CI: Confidence Interval
CVD: Cardiovascular Diseases
IQR: Interquartile Range
MHI-5: Mental Health 5-Items
OR: Odds Ratio
SES: Socioeconomic Status
