# Supplementary material for: Associations of psychosocial factors, knowledge, attitudes and practices with hospitalizations in internal medicine divisions in different population groups in Israel
Source: Int J Equity Health. 2021 Apr 20;20:105. doi: 10.1186/s12939-021-01444-z (PMC8056509; doi:10.1186/s12939-021-01444-z)
Supplement: Supplementary file 1 — Additional file 1. [file 12939_2021_1444_MOESM1_ESM.docx]

**Supplemental Table 1: Utilization of healthcare services in Arabs and Jews aged ≥ 40 years with hypertension, diabetes or cardiovascular disease (n= 28,412)**

|  | **Jewish Men** | **Arab Men** | **Jewish Women** | **Arab Women** | **P value** |
| --- | --- | --- | --- | --- | --- |
| **Total** | 8750 | 4826 | 9202 | 5615 |  |
| **Internal medicine hospitalization, n (%)** | 1025 (11.7) | 736 (15.3) | 1075 (11.7) | 680 (12.1) |  |
| **Internal medicine emergency room visit, n (%)** | 686 (7.8) | 539 (11.2) | 879 (9.6) | 622 (11.1) | <0.001 |
| **Number of medical consultants, median (25^th^ percentile, 75^th^ percentile)** | 3 (1, 6) | 2 (0, 4) | 3 (1, 7) | 2 (1, 5) | <0.001 |
| **Consulted a specialist, any, n (%)** | 6716 (76.8) | 3418 (70.8) | 7436 (80.8) | 4274 (76.1) | <0.001 |
| **Consulted a diabetes specialist, n (%)** | 575 (6.6) | 446 (9.2) | 660 (7.2) | 614 (10.9) | <0.001 |
| **Consulted a cardiologist, n (%)** | 2466 (28.2) | 1121 (23.2) | 1850 (20.1) | 884 (15.7) | <0.001 |
| **Consulted a specialty in surgery, n (%)** | 2808 (32.1) | 990 (20.5) | 2580 (28.0) | 1020 (18.2) | <0.001 |

P value was obtained by chi square test and Fisher Exact test, as appropriate, for categorical variables and Mann-Whitney U test for continuous and discrete variables.

Utilization of health services as determined by Clalit Health Services database
