# Supplementary material for: Associations of psychosocial factors, knowledge, attitudes and practices with hospitalizations in internal medicine divisions in different population groups in Israel
Source: Int J Equity Health. 2021 Apr 20;20:105. doi: 10.1186/s12939-021-01444-z (PMC8056509; doi:10.1186/s12939-021-01444-z)
Supplement: Supplementary file 2 — Additional file 2. [file 12939_2021_1444_MOESM2_ESM.docx]

**Sampling method and sample size calculation**

| **Sampling scheme and study flow chart** | | | |  |
| --- | --- | --- | --- | --- |
| **Total: 28,393** | | | |  |
| **Not hospitalized N=24877** | | **Hospitalized, N=3516** | |  |
| **Jews N=15,852** | **Arabs N=9025** | **Jews N=2100** | **Arabs N=1416** | **Total** |
| N=250 | N=250 | N=125 | N=125 | Randomly selected |
| N=163 (65%) | N=172(69%) | N=85 (68%) | N=100 (80%) | Agreed |
| **Male N=7725** | **Male N=4090** | **Male N=1025** | **Male N=736** | **Males** |
| N=125 | N=125 | N=62 | N=62 | Randomly selected |
| N=82 (66%) | N=67 (54%) | N=41 (66%) | N=50 (81%) | Agreed |
| **Females N=4935** | **Females N=8127** | **Females N=1075** | **Females N=680** | **Females** |
| N=125 | N=125 | N=63 | N=63 | Randomly selected |
| N=81 (65%) | N=105 (84%) | N=44 (69%) | N=50 (79%) | Agreed |
| **N=520** | | | | Total agreed |

**Pre-planned sample size calculation as it appeared in the study protocol**

Sample size was calculated according to differences between users and non-users of health services, in education level i.e. a higher proportion of persons with less education (<12 years) is expected among users of health services.

- The frequency of less educated persons among users of health services was assumed as 50% and among non-users, 25% (i.e. the difference between cases and controls in the frequency of less educated persons is 25%).
- Type one error 5%, power 80%.
- Compliance rate of 75%

Based on these assumptions the minimal sample size needed was 154 (77 in each group). Since separate analyses were planned according to ethnic groups, the required sample size was 616.
