# Supplementary material for: Associations of psychosocial factors, knowledge, attitudes and practices with hospitalizations in internal medicine divisions in different population groups in Israel
Source: Int J Equity Health. 2021 Apr 20;20:105. doi: 10.1186/s12939-021-01444-z (PMC8056509; doi:10.1186/s12939-021-01444-z)
Supplement: Supplementary file 3 — Additional file 3. [file 12939_2021_1444_MOESM3_ESM.docx]

**Comparison of the hospitalizations and risk factors between Jewish and Arab adults with selected chronic diseases**

**Participant questionnaire**

**Part A**

Serial number: _______

Name of interviewer: __________

1. Name of participant: _______
2. Sex: 1. Male 2. Female
3. Age of birth: _________
4. Country of birth: _____ Age of immigration to Israel: _____
5. Father country of birth______
6. Home phone number
7. Town of residency
8. Are you: 1. Jewish 2. Arab: a. Muslim b. Christian c. Druze 3. Other 4. Refuse to answer
9. Name of your general physician (GP): ______ what is your clinic branch: ______
10. Do you have complementary insurance? 1. No 2. Yes- private, elaborate which______

| 1. What is your marital status? 2. Married/in a relationship 3. Divorced 4. Widowed 5. Single 6. Refuse to answer | 1. Do you have children? 2. No 3. Yes, how many ____ 4. Refuse to answer |
| --- | --- |
| 1. How do you define yourself mainly- choose one answer? 2. Hired 3. Independent 4. Unemployed 5. Retired 6. House wife 7. Refuse to answer | 1. What was your main occupation for the last 5 years? ______ 2. If retired what was your main occupation most of your life? __________ |
| 1. What is your highest diploma? 2. High school diploma 3. Professional diploma 4. Academic diploma 1^st^/2^nd^/3^rd^ degree 5. Other- please specify____ 6. No diploma 7. Refuse to answer | 1. How many years have you studied? ______ 2. Do you know how to read and write in your mother tongue?   1. No 2. Yes.   1. Do you know how to read and write in Hebrew? 1. No 2. Yes. |
| 1. How do you define yourself? 2. Secular 3. Traditional 4. Religious 5. Ultra-orthodox 6. Refuse to answer | 1. What is your house average net income (including pension etc.)? 2. Less than 1700₪ 3. 1700-3600₪ 4. 3601-5200₪ 5. 5201-6500₪ 6. 6501-8500₪ 7. 8501-10,000₪ 8. Above 10,000₪ 9. Refuse to answer |
| 1. What is the number of rooms in your house (including living room excluding the kitchen and balcony)? _________ |  |
| 1. How many people live in your house in most of the week days? _______ |  |

**Part B: Health status**

1. In general, what is your health status
2. Very good
3. Good
4. Average
5. Quite bad
6. Very bad
7. In the last two weeks have you decreased activity of thing you normally do (at home work or in your free time) because of an injury or disease? 1. No 2. Yes
8. Do you have a chronic physical restriction that makes it difficult for you to complete everyday life activities as moving, eating, getting dressed etc’ (chronic = more than 6 months)? 1. No 2. Yes.
9. **Have you ever been diagnosed with one of the following diseases?**

Hypertension 1. No 2. Yes. 3. Don’t know 4. Refuse to answer

Diabetes 1. No 2. Yes. 3. Don’t know 4. Refuse to answer

Coronary heart disease 1. No 2. Yes. 3. Don’t know 4. Refuse to answer

Cerebrovascular Attack 1. No 2. Yes. 3. Don’t know 4. Refuse to answer

Heart failure 1. No 2. Yes. 3. Don’t know 4. Refuse to answer

Arrhythmia 1. No 2. Yes. 3. Don’t know 4. Refuse to answer

Dyslipidemia 1. No 2. Yes. 3. Don’t know 4. Refuse to answer

Asthma 1. No 2. Yes. 3. Don’t know 4. Refuse to answer

Osteoporosis 1. No 2. Yes. 3. Don’t know 4. Refuse to answer

Anxiety or depression 1. No 2. Yes. 3. Don’t know 4. Refuse to answer

Cancer 1. No 2. Yes. 3. Don’t know 4. Refuse to answer

Other diseases 1. No 2. Yes. Elaborate_____

**Part C: Prescription drug use**

1. Have you taken any prescribed drugs in the last two weeks: 1. No. 2. Yes.
2. **For which of the following diseases:**

Hypertension 1. No 2. Yes. 3. Don’t know 4. Refuse to answer

Diabetes 1. No 2. Yes. 3. Don’t know 4. Refuse to answer

Heart or vascular disease 1. No 2. Yes. 3. Don’t know 4. Refuse to answer

Dyslipidemia 1. No 2. Yes. 3. Don’t know 4. Refuse to answer

Asthma 1. No 2. Yes. 3. Don’t know 4. Refuse to answer

Anxiety or depression 1. No 2. Yes. 3. Don’t know 4. Refuse to answer

Other diseases Elaborate_____

1. Have you received a prescription from your GP in the last two weeks and didn’t buy the drug? 1. No. 2. Yes- why:
2. The drug was to expensive
3. No need for the drug anymore
4. Other _______

**Part 4- health services use**

**Physician visits and extra services**

1. For the last 4 weeks how many times have you visited your GP ? ____
2. When was your last visit of your GP?
3. In the last month
4. More than a month but less than 2
5. More than 2 months but less than 6
6. Before more than 6 months
7. Don’t remember
8. Refuse to answer
9. The last time you visited your GP what was the main reason?
10. Accident or injury
11. Disease or acute health problem
12. Regular check up
13. Prescription renewal
14. Administration
15. Other ___________
16. For the last 4 weeks how many times have visited a specialist or a surgeon regarding your health? ______
17. In how many of your visit for your GP did you receive a referral for a specialist?
18. Every visit
19. In half or more of the visit
20. Less than half of the visits
21. Never
22. When was your last visit of a specialist or a surgeon?
23. In the last month
24. More than a month but less than 2
25. More than 2 months but less than 6
26. Before more than 6 months
27. Don’t remember
28. Refuse to answer
29. Last time you visited a specialist or a surgeon what was the main reason?
30. Accident or injury
31. Disease or acute health problem
32. Regular check up
33. Prescription renewal
34. Administration
35. Other ___________
36. How much money did you spend out of pocket in the last month for physician visits? ____
37. Are you being monitors by one of the following specialists or in the clinic for (1 or more answers): 1. Diabetes 2. Cardiologist 3. Hypertension 4. Other _____
38. For the last 12 month have you used one of the following health services:
39. Physiotherapy 1. No 2. Yes.
40. Dietician 1. No 2. Yes.
41. Mental health clinic 1. No 2. Yes.
42. Complementary medicine 1. No 2. Yes.
43. Other ______
44. Have you ever done one of the following examinations:

| Occult blood | No | Yes, how many years ago____ | Refuse to answer |
| --- | --- | --- | --- |
| Colonoscopy | No | Yes, how many years ago____ | Refuse to answer |
| Mammography (Women) | No | Yes, how many years ago____ | Refuse to answer |
| Pap smear (Women) | No | Yes, how many years ago____ | Refuse to answer |
| Bone density scan | No | Yes, how many years ago____ | Refuse to answer |
| PSA (Men) | No | Yes, how many years ago____ | Refuse to answer |

**Visits in the Emergency room (ER) and hospitalizations**

1. For the last 12 month have you visited the ER and have not been hospitalized in that visit:
2. No 2. Yes- which: 1. Internal 2. Orthopedics 3. Gynecology 4. Surgery.
3. How many times have you visited the ER in the last 12 months? ______
4. Of these visits, in how many did you receive a referral from your GP?
5. Every time
6. Half or more
7. Less than half
8. In none of my visits
9. Are you aware that visiting the ER without a referral costs private money? 1. No. 2. Yes.
10. How much money did you spend out of pocket for ER visits for the last year? ______
11. For the last 12 month, have you been hospitalized in the hospital for one night or more: 1. No 2. Yes- which department in the last hospitalization? ________
12. How many times have you been hospitalized for the 12month? ________
13. Of these hospitalization, in how many times did you receive a referral from your GP?
14. Every time
15. Half or more
16. Less than half
17. In none of my visits
18. What was the reason for your last hospitalization?
19. Accident or injury
20. Disease or acute health problem
21. Elective surgery
22. Other __________
23. How much money did you spend out of pocket for hospitalizations for the last year? ______

**Part E- Barriers in usage of health services**

| 1. How would you define your Hebrew fluency? 2. Very good 3. Good 4. Moderate 5. Weak 6. Very weak | 1. How much do you need translation while hospitalized or visiting the emergency room? 2. Very much 3. Moderately 4. Slightly 5. Not at all |
| --- | --- |
| 1. How much do you need translation while visiting a specialist? 2. Very much 3. Moderately 4. Slightly 5. Not at all | 1. How would you define the difficulty arriving to the hospital? 2. Very high 3. high 4. Moderate 5. Low 6. Not at all |
| 1. How would you define the difficulty arriving to a specialist? 2. Very high 3. high 4. Moderate 5. Low 6. Not at all | 1. How do you usually arrive to the hospital? 2. By private car 3. By ambulance 4. By public transportation 5. By car rental 6. Other ____ |
| 1. How do you usually arrive to a specialist? 2. By private car 3. By public transportation 4. By car rental 5. Other ____ |  |

**Part F- Mental health and health practices**

| For the 4 weeks, how often did you feel: | | | | | | | |
| --- | --- | --- | --- | --- | --- | --- | --- |
|  | All the time | Most of the time | Very often | Sometimes | Rarely | Never | Refuse to answer |
| 1. Very stressed or tensed | 1 | 2 | 3 | 4 | 5 | 6 | 7 |
| 1. Bad mood that nothing can cheer me up | 1 | 2 | 3 | 4 | 5 | 6 | 7 |
| 1. Calm and peaceful | 1 | 2 | 3 | 4 | 5 | 6 | 7 |
| 1. Desperate or depressed | 1 | 2 | 3 | 4 | 5 | 6 | 7 |
| 1. Happy | 1 | 2 | 3 | 4 | 5 | 6 | 7 |

1. How many people are close to you that you can rely on when you have serious problems?
2. None
3. 1-2
4. 3-5
5. More than 6
6. Don’t know
7. Refuse to answer
8. How much caring, interest, and worries do people show in what you do?
9. A lot
10. Moderately
11. A bit
12. Not at all
13. Refuse to answer
14. How easy is it to get practical help from your neighbors when you need it?
15. Very easy
16. Easy
17. Possible
18. Hard
19. Very hard
20. Don’t know
21. Refuse to answer

9. Do you usually practice sports as: walking, swimming, running, gymnastics, bicycle or ball games for at least 20 minutes?

1. Almost every day
2. Once or twice a week
3. Once or twice a month
4. Less than once a month
5. Never

10. Do you smoke: 1. No 2. Smoked in the past 3. Yes.

11. What do you smoke? 1. Cigarettes 2. Cigars 3. Pipe 4. Shisha

12. How many cigarettes do you smoke a day? ________

13. What age did you start smoking? ______

14. In the last 4 weeks have you drunk an alcoholic beverage? 1. No 2. Yes, how many times? __

15. What is your height without shoes? _____(Meters) 2. Don’t know 3. Refuse to answer

16. What is your weight with light close and no shoes? __(Kg) 2. Don’t know 3. Refuse to answer

**Part G- Knowledge and perceptions**

What of the following sentences is correct/not correct

1. No need to change your diet because of high blood pressure. 1. Wrong 2. Correct
2. High blood pressure might cause stroke 1. Wrong 2. Correct
3. Good dietary habits might prevent diabetes or heart diseases 1. Wrong 2. Correct
4. High blood pressure isn’t a risk factor to a heart attack 1. Wrong 2. Correct
5. Diuretics decrease blood pressure 1. Wrong 2. Correct
6. The reason for diabetes is a liver problem 1. Wrong 2. Correct
7. Obesity is a cause for diabetes 1. Wrong 2. Correct
8. Diabetes is an incurable disease 1. Wrong 2. Correct
9. Diabetes in adults should be treated by insulin and not pills 1. Wrong 2. Correct
10. Diabetes could cause back pain 1. Wrong 2. Correct
11. Physical activity is important in disease prevention 1. Wrong 2. Correct
12. Diabetes could harm the eyes 1. Wrong 2. Correct
13. Heart attack is caused by a thrombus in the arteries 1. Wrong 2. Correct
14. Smoking does not cause heart attacks 1. Wrong 2. Correct
15. Physical activity does not lower the risk of heart attacks 1. Wrong 2. Correct
16. Aspirin can lower the risk of heart attacks 1. Wrong 2. Correct

Now I will present you with sentences regarding prevention and treatment of chronic diseases. There is nothing right or wrong, the answer is based on how you feel about these questions.

Please specify in what degree the statement fits what you feel:

1. Very much
2. Fits well
3. Doesn’t fit to much
4. Doesn’t fit at all

| **Perceptions:** | 1 | 2 | 3 | 4 |
| --- | --- | --- | --- | --- |
| 1. Examinations for early detection are important to decrease the disease damage |  |  |  |  |
| 1. There is no way to prevent disease it is all in the hand of god |  |  |  |  |
| 1. All the time there are new exams and medicines and one should use them |  |  |  |  |
| 1. You shouldn’t do to many exams the fate will decide |  |  |  |  |
| 1. Diseases are a punishment for walking away from religion |  |  |  |  |
| 1. Complementary medicine helps more treating diseases |  |  |  |  |
| 1. Its true you should accept faith but you can relieve the damage of diseases |  |  |  |  |
| 1. I trust the knowledge of my general practitioner |  |  |  |  |
| 1. In the clinic I get the best care |  |  |  |  |
| 1. I feel as a burden on my family with all of my exams |  |  |  |  |
| 1. Routine checkup with your general practitioner is important to decrease the severity of your disease |  |  |  |  |
| 1. It is very easy to organize an exam with a health specialist |  |  |  |  |
| 1. It is hard for me with all the expenses on exams and medications |  |  |  |  |
| 1. For women: sometimes I am sick and I don’t go to my general practitioner because he is a man |  |  |  |  |
| 1. Complementary medicine is better than western medicine in many things |  |  |  |  |
| 1. Patients who use traditional medicine jeopardize their health |  |  |  |  |
| 1. I don’t trust the knowledge and perception of physicians and the health ministry |  |  |  |  |
| 1. Only spiritual healers or Sheih’s can heal diseases |  |  |  |  |
| 1. Disease are caused by the devil’s eye |  |  |  |  |

**Information materials**

Please mention if you received from a health professional

1. Information materials on your disease in your mother tongue: 1. No. 2. Yes.
2. Face to face teaching regarding diet, physical activity, leg treatment: 1. No. 2. Yes.
3. A workshop regarding diabetes/ hypertension etc: 1. No. 2. Yes.

**PART H- Exposure to traumatic events**

1. Have you ever been in a severe traumatic event as an car accident, a disease risking your life, a terror attack an assault or anything traumatic else?
2. No 2. Yes, how long ago? ____ months ____ years
3. Have you experiences one of the following events in the last 12 months:

|  | 1. **No** | 1. **Yes** | 1. **Refuse** |
| --- | --- | --- | --- |
| An additional family member or a family member leaving the house (include birth/adoption/new relationship, not including death) |  |  |  |
| A severe disease or injury |  |  |  |
| Death of a family member |  |  |  |
| Death of a close friend |  |  |  |

3. Have you ever lived under military rule? 1. No 2. Yes, when? _____

4. Have you or your family ever had to leave your house or escape to a different country or place? 1. No 2. Yes, when _____

5. Have you or your family loose property as a house or lands while war? 1. No 2. Yes, when? _

6. Are you part of a national minority group? 1. No 2. Yes
