# Supplementary material for: Associations of psychosocial factors, knowledge, attitudes and practices with hospitalizations in internal medicine divisions in different population groups in Israel
Source: Int J Equity Health. 2021 Apr 20;20:105. doi: 10.1186/s12939-021-01444-z (PMC8056509; doi:10.1186/s12939-021-01444-z)
Supplement: Supplementary file 4 — Additional file 4. [file 12939_2021_1444_MOESM4_ESM.docx]

| Detailed operational definitions of all study variables | | | |
| --- | --- | --- | --- |
| Variables | **Variable type** | **Additional data** | **Question number** |
| Socio-demographic variables | | | |
| Sex | Dichotomous: males, females | Based on Clalit database records |  |
| Population group | Dichotomous: Arab or Jews |  | A8 |
| Age, years | Continuous | Based Clalit database records |  |
| Country of birth | Categorical: Israel, Asia/Africa, or Europe/America/ Russia | Participants' report | A4 |
| Marital status, not married | Dichotomous, yes=1, no=0 | Not married includes single, divorced, widowed, based on the participants' report | A11 |
| Number of children | Discrete |  | A12 |
| Household density | Composite variable, Continuous | Number of rooms divided to number of people living in the house | A22-23 |
| Monthly income above 3600 Shekels, | Dichotomous, yes=1, no=0 | Participants' report | A21 |
| Religious, yes | Dichotomous, yes=1, no=0 | Yes=Religious or orthodox  No= Secular or traditional | A20 |
| Years of schooling | Discrete | Participants' report | A17 |
| Health status and lifestyle | | | |
| Comorbidity score | Composite variable, discrete | Based on diagnosis in Clalit database (1) |  |
| Mental health items 5 (MHI-5) | Composite variable, continuous variable | (2-6) | F1-5 |
| Self-rated health, not good | Dichotomous, yes=1, no=0 | Yes=Intermediate or less  No= Very good or good | B1 |
| Physical inactivity | Dichotomous, yes=1, no=0 | Yes=physically inactive  No=physical activity at least 20 minutes, in any frequency | F9 |
| Body mass index | Composite variable, Continuous | Weight (kg)/ height (m) ^2^  Participants' reports on weight and height | F15-16 |
| Smoking | Dichotomous, yes=1, no=0 | No includes never smoker and past smoker. Participants' report | F10 |
| Access and barriers to healthcare services | | | |
| Complementary health insurance | Dichotomous, yes=1, no=0 | Z score was calculated using factor analysis and categorized by the median | A10 |
| Fluency in Hebrew/ needs translation in medical interaction | Categorical: a score below the median level vs. a score equal to or higher than the median | Z score was calculated using factor analysis and categorized by the median level. | E1, E2, E3 |
| Perceived treatment difficulties and burden | Categorical: a score below the median level vs. a score equal to or higher than the median | Z score was calculated using factor analysis and categorized by the median | H10, H13 |
| Health-related knowledge and attitudes/ Beliefs | | | |
| Knowledge regarding health & lifestyle risk factors | Continuous variable | Z score was calculated using factor analysis and categorized by the median | G2, G3, G7, G11, G12, G13 |
| Believes in alternative medicine | Categorical: a score below the median level vs. a score equal to or higher than the median | Z score was calculated using factor analysis and categorized by the median | H6, H15 |
| Believes in superstition and fate | Categorical: a score below the median level vs. a score equal to or higher than the median | Z score was calculated using factor analysis and categorized by the median. | H2, H4, H5, H19 |
| Trust in medical system and importance of preventive medicine | Categorical: a score below the median level vs. a score equal to or higher than the median | Z score was calculated using factor analysis and categorized by the median level. Higher values represent higher trust | H1, H3, H8, H9, H11 |

**References**

1. Sagie S, Naamnih W, Frej J, Cohen D, Alpert G, Muhsen K. Correlates of hospitalizations in internal medicine divisions among Israeli adults of different ethnic groups with hypertension, diabetes and cardiovascular diseases. Plos One. 2019;14(4).

2. Rumpf HJ, Meyer C, Hapke U, John U. Screening for mental health: validity of the MHI-5 using DSM-IV Axis I psychiatric disorders as gold standard. Psychiatry Res. 2001;105(3):243-53.

3. McCabe CJ, Thomas KJ, Brazier JE, Coleman P. Measuring the mental health status of a population: a comparison of the GHQ-12 and the SF-36 (MHI-5). Br J Psychiatry. 1996;169(4):516-21.

4. Meltzer H. Development of a common instrument of mental health. In: Nosikov A, Gudex C, editors. EUROHIS Developing Common Instruments for Health Surveys. Amsterdam: IOS Press; 2003. p. 35-49.

5. Muhsen K, Garty-Sandalon N, Gross R, Green MS. Psychological distress is independently associated with physical inactivity in Israeli adults. Prev Med. 2010;50(3):118-22.

6. Ware JE, Jr., Sherbourne CD. The MOS 36-item short-form health survey (SF-36). I. Conceptual framework and item selection. Med Care. 1992;30(6):473-83.

| Factor analysis |
| --- |
| Confirmatory factor analysis Fluency in Hebrew/ needs translation in medical interaction |
| A confirmatory factor analysis with varimax rotation was conducted. Coefficients with absolute value below 0.4 were excluded. The factors analysis included in the analysis the following 3 items (Part E in the questionnaire- E1, E2, E3).  How would you define your Hebrew fluency?   1. Very good 2. Good 3. Moderate 4. Weak 5. Very weak   How much do you need translation while hospitalized or visiting the emergency room?  1. Very much 2. Moderately 3. Slightly 4. Not at all  How much do you need translation while visiting a specialist?  1. Very much 2. Moderately 3. Slightly 4. Not at all  This resulted in 1 component with Eigen value >1.0 that explained 69.4% of the variance. Alpha Cronbach was 0.83 |
| Exploratory factor analysis-1: Health-related knowledge |
| An exploratory factor analysis with varimax rotation was conducted. Coefficients with absolute value below 0.4 were excluded. The factors analysis included in the analysis the following 14 items (Part G in the questionnaire).  G1: No need to change your diet because of high blood pressure  G2: High blood pressure might cause stroke  G3: Good dietary habits might prevent diabetes or heart diseases  G4: High blood pressure isn’t a risk factor to a heart attack  G5: Diuretics decrease blood pressure  G6: The reason for diabetes is a liver problem  G7: Obesity is a cause for diabetes  G8: Diabetes is an incurable disease  G11: Physical activity is important in disease prevention  G12: Diabetes could harm the eyes  G13: Heart attack is caused by a thrombus in the arteries  G14: Smoking does not cause heart attacks  G15: Physical activity does not lower the risk of heart attacks  G16: Aspirin can lower the risk of heart attacks  The Kaiser-Meyer-Olkin Measure of Sampling Adequacy of this analysis was 0.74. P value <0.001 by the Bartlett’s Test of Sphericity, thus suggesting that the factor analysis was adequate.  This resulted in 4 components with Eigen value >1.0 that explained 44.7% of the variance, of those one factor with 6 items G2, G3, G7, G11, G12, G13 had the highest Eigen value of 2.5 and explained 17.9% of the variance, represented a reasonable score of "knowledge on health and lifestyle risk factors". Alpha Cronbach of all 6-items included in this scale was 0.66 |
| Exploratory factor analysis-2: Health-related attitudes and believes |
| An exploratory factor analysis with varimax rotation was conducted. Coefficients with absolute value below 0.4 were excluded. The factors analysis included in the analysis the following 13 items (Part H in the questionnaire).  H1: Examinations for early detection are important to decrease the disease damage  H2: There is no way to prevent disease it is all in the hand of God  H3: All the time there are new exams and medicines and one should use them  H4: You shouldn’t do to many exams the fate will decide  H5: Diseases are a punishment for walking away from religion  H6: Complementary medicine helps more treating diseases  H8: I trust the knowledge of my general practitioner  H9: In the clinic I get the best care  H10: I feel as a burden on my family with all of my exams  H11: Routine checkup with your general practitioner is important to decrease the severity of your disease  H13: It is hard for me with all the expenses on exams and medications  H15: Complementary medicine is better than western medicine in many things  H19: Disease are caused by the devil’s eye  The Kaiser-Meyer-Olkin Measure of Sampling Adequacy of this analysis was 0.65. P value <0.001 by the Bartlett’s Test of Sphericity, thus suggesting that the factor analysis was adequate.  This resulted in 4 components with Eigen value >1.0 that explained 52.7% of the variance, as following  "Trust in medical system and importance of preventive medicine" (5 items, H1, H3, H8, H9, H11) Alpha Cronbach 0.67  "Believes in superstition and fate" (4 items H2, H4, H5, H19) Alpha Cronbach 0.61  "Believes in alternative medicine" (2 items, H6, H15). Alpha Cronbach 0.52  "Perceived treatment difficulties and burden" (2 items, H10, H13). Alpha Cronbach 0.38 |
