# Supplementary material for: Associations of psychosocial factors, knowledge, attitudes and practices with hospitalizations in internal medicine divisions in different population groups in Israel
Source: Int J Equity Health. 2021 Apr 20;20:105. doi: 10.1186/s12939-021-01444-z (PMC8056509; doi:10.1186/s12939-021-01444-z)
Supplement: Supplementary file 5 — Additional file 5. [file 12939_2021_1444_MOESM5_ESM.docx]

Missing values for each independent variables

| **Statistics** | | |  |
| --- | --- | --- | --- |
|  | **Number** | |  |
| **Variable** | **Valid** | **Missing** | **% Missing values** |
| Sex | 520 | 0 | 0.00 |
| Age | 518 | 0 | 0.00 |
| Population | 520 | 0 | 0.00 |
| Marital status | 520 | 0 | 0.00 |
| Number of offspring | 520 | 0 | 0.00 |
| Household density | 513 | 7 | 1.36 |
| Income | 424 | 96 | 22.64 |
| Religiosity | 510 | 10 | 1.96 |
| Years of schooling | 520 | 0 | 0.00 |
| Comorbidity score | 520 | 0 | 0.00 |
| hypertension | 520 | 0 | 0.00 |
| cardiovascular disease | 520 | 0 | 0.00 |
| diabetes | 520 | 0 | 0.00 |
| Mental health 5 items | 518 | 2 | 0.39 |
| Self-rated health status | 519 | 1 | 0.19 |
| Physical inactivity | 512 | 8 | 1.56 |
| Body mass index | 492 | 28 | 5.69 |
| Smoking | 519 | 1 | 0.19 |
| Complementary health insurance | 480 | 40 | 8.33 |
| Trust in medical system and importance of prevention | 520 | 0 | 0.00 |
| Believes in fate & superstition | 520 | 0 | 0.00 |
| Believes in alternative medicine | 520 | 0 | 0.00 |
| Perceived difficulty and burden | 520 | 0 | 0.00 |
| Low Hebrew fluency, needs translation in medical interaction | 520 | 0 | 0.00 |
| Knowledge in health and lifestyle risk factors | 520 | 0 | 0.00 |

Interactions between the variable "population group" with sociodemographic and health-related variables in the association with correlates of hospitalizations in internal medicine divisions

|  | **OR (95% CI)** | **P value** |
| --- | --- | --- |
| Sex | 0.64 (0.39-1.05) | 0.08 |
| Population group | 0.39 (0.12-1.26) | 0.1 |
| Population group by sex | 1.70 (0.83-3.51) | 0.2 |
| Age | 1.02 (1.00-1.05) | 0.04 |
| Population group | 0.85 (0.08-8.76) | 0.9 |
| Population group by age | 0.99 (0.96-1.03) | 0.9 |
| Marital status | 1.12 (0.64-1.98) | 0.7 |
| Population group | 0.63 (0.21-1.87) | 0.4 |
| Population group by marital status | 1.28 (0.59-2.81) | 0.5 |
| Number of offspring | 1.08 (0.99-1.18) | 0.1 |
| Population group | 0.73 (0.34-1.59) | 0.4 |
| Population group by number of offspring | 1.10 (0.94-1.28) | 0.2 |
| Household density | 0.99 (0.68-1.44) | 1.0 |
| Population group | 0.84 (0.39-1.82) | 0.7 |
| Population group by household density | 1.16 (0.48-2.82) | 0.8 |
| Monthly income | 0.69 (0.40-1.21) | 0.2 |
| Population group | 1.22 (0.69-2.14) | 0.5 |
| Population group by monthly income | 0.71 (0.32-1.60) | 0.4 |
| Religiosity | 0.99 (0.58-1.69) | 0.2 |
| Population group | 0.70 (0.20-2.43) | 0.7 |
| Population group by religiosity | 1.25 (0.55-2.87) | 0.8 |
| Number of years of schooling | 0.97 (0.92-1.01) | 0.2 |
| Population group | 1.38 (0.71-2.66) | 0.3 |
| Population group by number of years of schooling | 0.97 (0.90-1.04) | 0.3 |
| Comorbidity score | 1.57 (1.33-1.87) | <0.001 |
| Population group | 1.09 (0.36-3.27) | 0.9 |
| Population group by comorbidity score | 0.96 (0.75-1.23) | 0.7 |
| Mental health index (MHI-5 score | 0.97 (0.95-0.99) | 0.001 |
| Population group | 1.58 (0.28-8.92) | 0.6 |
| Population group by MHI-5 score | 0.99 (0.96-1.02) | 0.6 |
| Self-rated health status | 4.48 (2.32-8.65) | <0.001 |
| Population group | 1.31 (0.58-2.93) | 0.5 |
| Population group by self-rated health status | 0.63 (0.25-1.56) | 0.3 |
| Physical inactivity | 2.12 (1.25-3.58) | 0.005 |
| Population group | 0.82 (0.45-1.49) | 0.5 |
| Population group by physical inactivity | 1.28 (0.60-2.74) | 0.5 |
| BMI | 1.00 (0.96-1.05) | 0.9 |
| Population group | 0.39 (0.05-2.98) | 0.4 |
| Population group by body mass index | 1.03 (0.96-1.10) | 0.4 |
| Smoking | 1.74 (1.00-3.04) | 0.05 |
| Population group | 1.64 (0.51-5.26) | 0.4 |
| Population group by smoking | 0.63 (0.25-1.59) | 0.3 |
| Complementary health insurance | 1.13 (0.65-1.95) | 0.6 |
| Population group | 1.61 (0.76-3.42) | 0.2 |
| Population group by complementary health insurance | 0.48 (0.19-1.16) | 0.1 |
| Low Hebrew fluency, needs translation in medical interaction | 0.88 (0.71-1.10) | 0.3 |
| Population group | 0.99 (0.68-1.43) | 0.9 |
| Population group by low Hebrew fluency, needs translation in medical interaction | 0.90 (0.61-1.33) | 0.6 |
| Perceived difficulty & burden | 1.42 (1.09-1.85) | 0.01 |
| Population group | 0.88 (0.60-1.27) | 0.5 |
| Perceived difficulty & burden | 1.23 (0.83-1.81) | 0.3 |
| Knowledge in health and lifestyle risk factors | 0.86 (0.64-1.16) | 0.3 |
| Population group | 0.86 (0.59-1.23) | 0.4 |
| Population group by knowledge in health and lifestyle risk factors | 1.01 (0.70-1.47) | 0.9 |
| Trust in medical system and importance of prevention | 0.90 (0.72-1.13) | 0.4 |
| Population group | 0.91 (0.63-1.31) | 0.6 |
| Population group by trust in medical system and importance of prevention | 1.09 (0.76-1.58) | 0.6 |
| Believes in alternative medicine | 0.79 (0.58-1.07) | 0.1 |
| Population group | 0.93 (0.64-1.35) | 0.7 |
| Population group by believes in alternative medicine | 1.36 (0.93-2.00) | 0.1 |
| Believes in fate & superstition | 0.84 (0.63-1.13) | 0.3 |
| Population group | 0.90 (0.62-1.30) | 0.6 |
| Population group by believes in fate & superstition | 1.43 (0.98-2.09) | 0.06 |

BMI: body mass index MHI-5: mental health item

Interactions were assessed in logistic regression models; each model included the independent variable "population group" as main effect, one additional independent variable of interest (e.g., age, sex, etc.) and an interaction term between the population group and the other independent variable included in the model

Multivariable analysis of the correlates of hospitalizations in internal medicine divisions

|  | **Adjusted OR** **[95% CI]** | **P** |
| --- | --- | --- |
| Population group (Jews vs. Arab) | 1.18 [0.76 -1.83] | 0.5 |
| Number of offspring | 1.08 [0.99-1.16] | 0.07 |
| Comorbidity score | 1.41 [1.24-1.61] | <0.001 |
| Physical inactivity | 1.41 [0.91-2.18] | 0.1 |
| Perceived difficulty & burden | 1.18 [0.94-1.48] | 0.2 |
| Subjective health status | 1.90 [1.14-3.16] | 0.01 |
| MHI-5 score | 0.97 [0.96-0.99] | 0.004 |
| Believes in fate & superstition | 0.87 [0.63-1.20] | 0.4 |
| Believes in fate & superstition by population group | 1.11 [0.73-1.71] | 0.6 |

CVD: cardiovascular disease, MHI-5: mental health items. Model R^2^ Nagelkerke=0.23

Multivariable analysis of the correlates of hospitalizations in internal medicine divisions

|  |  | |
| --- | --- | --- |
|  | Adjusted OR [95% CI] | P |
| Population group (Jews vs. Arab) | 1.31 [0.78-2.22] | 0.3 |
| Number of offspring | 1.07 [0.99-1.16] | 0.1 |
| Comorbidity score | 1.38 [1.21-1.58] | <0.001 |
| Physical inactivity | 1.34 [0.86-2.08] | 0.2 |
| Perceived difficulty & burden | 1.19 [0.95-1.49] | 0.1 |
| Subjective health status | 1.98 [1.17-3.34] | 0.01 |
| MHI-5 score | 0.98 [0.96-0.99] | 0.006 |
| Monthly income (imputed data) | 0.88 [0.53-1.46] | 0.6 |
| Sex | 0.69 [0.44-1.08] | 0.1 |
| Age | 0.99 [0.98-1.02] | 0.8 |
| Number of years of schooling | 0.99 [0.94-1.04] | 0.6 |

CVD: cardiovascular disease, MHI-5: mental health items. Model R^2^ Nagelkerke=0.24
